# Effect of Heat Treatment on In Vitro Cytotoxicity of Ti-Nb-Zr Gum Metal Alloy

**DOI:** 10.3390/ma18194473

**Published:** 2025-09-25

**Authors:** Arash Etemad, Saeed Hasani, Alireza Mashreghi, Fariba Heidari, Parinaz Salehikahrizsangi, Sabine Schwarz, Katarzyna Bloch, Marcin Nabialek

**Affiliations:** 1 Department of Mining and Metallurgical Engineering, Yazd University, Yazd 8915818411, Iran; etemadarash1@gmail.com (A.E.); hasani@yazd.ac.ir (S.H.); amashreghi@yazd.ac.ir (A.M.); 2 Dental Research Center, Dental Research Institute, School of Dentistry, Isfahan University of Medical Sciences, Isfahan 8174673461, Iran; fariba.heidari77@gmail.com; 3 University Service Centre for Transmission Electron Microscopy, Technische Universität Wien, Wiedner Hauptstrasse 8-10, 1040 Wien, Austria; sabine.schwarz@tuwien.ac.at; 4 Department of Physics, Częstochowa University of Technology, 42-201 Częstochowa, Poland; katarzyna.bloch@pcz.pl (K.B.); nmarcell@wp.pl (M.N.)

**Keywords:** titanium-based biomaterials, gum metal, MG-63 cells, in vitro cytotoxicity, phase transformation, cytocompatibility

## Abstract

Strain-induced deformations and phase evolutions are two hidden factors that may influence cytocompatibility of Gum Metal alloys during processing for relevant implant applications. In the present research, changes in cell viability of a new Gum Metal Ti-Nb-Zr alloy in its cold-rolled state and after heat treatments (at 700, 850, and 900 °C) were investigated by a comprehensive study of microstructural phases and their role in deformation mechanisms as well as mechanical properties. In its cold-rolled state, the alloy showed a lamellar microstructure along with stress-induced *α*″ martensite and *ω* phases, as confirmed by optical microscopy (OM) and X-ray diffractometry (XRD) analysis. The instability in the *β* phase led to a strain-induced martensitic (SIM) transformation from *β* to *α*′/*α*″ phases, causing lower viability of MG-63 cells compared with commercially pure titanium. MG-63 cell viability was significantly higher (*p* < 0.0001) in the alloy heat-treated at 900 °C compared with those heat-treated at 700 and 850 °C. This can be directly attributed to the increased portion of the stable and dominant *β* phase. The stabilized *β* phase greatly improved the alloy’s cellular response by reducing harmful phase interactions and maintaining mechanical compatibility with bone (admissible strain of 1.3%). Importantly, heat treatment at high temperatures (between 850 and 900 °C) effectively converted the stress-induced *α*″ and *ω* phases back into a stable *β* phase matrix as the dominant phase.

## 1. Introduction

Metastable *β*-titanium alloys, characterized by a body-centered cubic (bcc) *β* phase, enjoy a favorable combination of low elastic modulus, good corrosion resistance, and biocompatibility, which makes them promising candidates for load-bearing implants that better match the mechanical behavior of natural bone, reducing stress shielding and improving patient outcomes [1,2,3,4]. The metastable nature of these alloys allows for phase transformations and microstructural modifications through heat treatment, which critically influence their mechanical behavior and bioactivity [5,6].

Heat treatment is followed by quenching, which results in precipitation of the *α* phase and other metastable phases (e.g., *α*′, *α*″, and *ω*) and changes the fraction of stabilized desirable *β* phase in the microstructure [5,7]. Linked to the mentioned phase transformations, heat treatment can minimize bone/implant modulus mismatch by optimizing the strength-ductility balance in metastable *β*-titanium alloys by providing for plasticity deformation mechanisms, i.e., transformation-induced plasticity (TRIP) and twinning-induced plasticity (TWIP) [6].

The above-mentioned microstructural changes and deformation-induced surface features can affect the biological response of metastable *β*-titanium alloys [8]. Specific phases, particularly the metastable *β* phase and deformation-induced martensitic *α*″ phase, play a critical role in determining mechanical behavior and surface characteristics that affect ion release and cell response. Enhancement in strain hardening and ductility in the presence of precipitated phases facilitates TWIP/TRIP mechanisms during deformation, leading to improved mechanical compatibility with bone. For MG-63 cells, improved cytocompatibility has been linked to microstructures that balance strength and ductility without excessive stiffness. Excessive *α*-phase precipitation and high modulus can impair cell adhesion and proliferation, while refined *β* grains and controlled plasticity mechanisms promote a more favorable surface environment for MG-63 cells [6].

In recent years, Gum Metals, based on the Ti-Nb-Zr system, have gained increasing attention for use in advanced implants. These novel alloys stand out for their exceptional mechanical properties, including an ultra-low elastic modulus, a high yield strength, and the rare ability to deform elasto-plastically without showing notable strain hardening. Thanks to their carefully designed chemical composition and the presence of a highly uniform, metastable *β* phase, these alloys exhibit pseudo-superelastic properties. This unique behavior enables better alignment with the natural flexibility of bone and helps minimize the negative effects of stress shielding [9,10,11,12].

While Gum Metal offers impressive mechanical properties, its biological performance is closely tied to its microstructure, especially after undergoing heat treatment. Thermal processes can lead to the formation of secondary phases, shift the stability of the *β* phase, and alter surface features. These changes may influence ion-release behavior, cellular cytotoxicity, and overall biocompatibility of the alloy. Although elements like Nb and Zr are generally considered biocompatible on their own, their combined behavior in a heat-treated alloy matrix is still not fully understood. That is why it is essential to evaluate these effects through in vitro studies, particularly using well-established cellular models such as MG-63 osteoblast-like cells [13,14,15].

Recent research has highlighted the importance of heat treatment and surface modification in improving the biocompatibility of Gum Metal alloys. For instance, studies on the Ti-36Nb-2Ta-3Zr-0.3O (Gum Metal) composition have shown that applying chemical and thermal treatments can produce nanoscale surface features. These nanostructures enhance biocompatibility and support better cell attachment and growth. This evidence indicates that Gum Metal is not just impressive in terms of its mechanical behavior; it also shows strong potential for positive cellular interactions. As a result, it stands out as a promising material for next-generation, smart biomedical implants [16,17].

In this research, a new Gum Metal alloy with a high allowable elastic strain, low Young’s modulus, and high strength and ductility is investigated. In case of cytocompatibility, it can be considered as a safer and more efficient alloy for conventional cases in bone (dental and orthopedic) applications. In this regard, the main aim of the present study is to elucidate the relationship between heat treatment temperatures, phase fraction evolution, and activation of TWIP/TRIP mechanisms in a newly designed *β* Ti alloy with a nominal composition of Ti-37.5Nb-4.5Zr, with a focus on their impact on cytocompatibility. Also, a detailed study of the behavior of *β*, *α*′, *α*″, and *ω* phases and their role in TWIP and TRIP mechanisms and hierarchical twinning systems is another main goal of this research, which can lead to a simultaneous increase in strength and ductility, a multifaceted view that has not been comprehensively considered in the scientific literature so far.

For this purpose, the cast ingots were cold-rolled up to 95% of reduction, followed by heat treatment at different temperatures (700, 850, and 900 °C) for 30 min and then quenched in iced water. Stable *β* phase and *α*′ and martensitic *α*″ phases were formed, influencing the alloy’s deformation response and surface characteristics. The resulting microstructural changes were correlated with cellular responses to provide new insights into the development of more biocompatible alloy designs for clinical applications.

## 2. Materials and Methods

### 2.1. Specimens Preparation

The Gum Metal Ti-Nb-Zr alloy ingot was synthesized using a vacuum arc remelting process to ensure homogeneity and control over the microstructure. The composition included specific weight percentages of titanium ingots (99.999%), zirconium ingots (99.95%), and niobium ingots (99.95%), all supplied by Exotech Incorporation (Pompano Beach, FL, USA). During the melting process, due to the arcing nature of the procedure and the small volume of the molten material, good turbulence is generated within the melt. This significantly contributes to the homogeneity of the produced alloy. After the melting process is complete, the easy separation of the molten charge from the crucible eliminates the need for any additional cleaning operations, leaving the crucible ready for the next charge.

**Figure 1:**
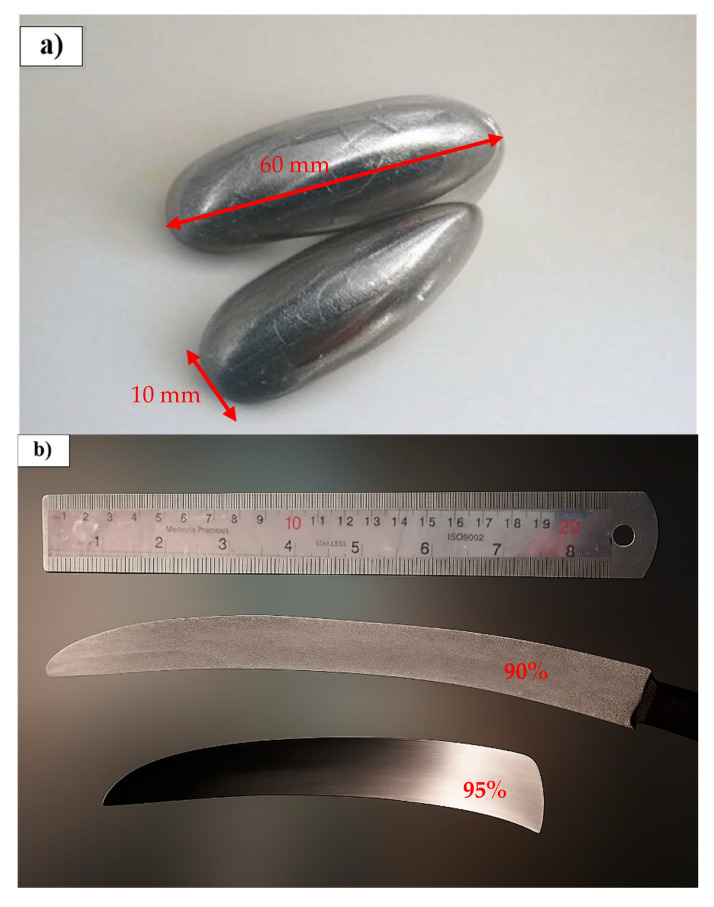
Process of Ti-Nb-Zr manufacturing: (**a**) alloy ingot and (**b**) after cold rolling to 90 and 95% of reduction in thickness.

The cast ingots had an almond-like shape as represented in Figure 1a, with an approximate mass of 30 g, a central diameter of about 10 mm, and a length of around 60 mm (Figure 1a). Specimens were cut from the obtained ingot using the Electrical Discharge Machine (EDM). Using Inductively Coupled Plasma Optical Emission Spectroscopy (ICP-OES) and infrared absorption with a Leco ONH836 analyzer, the composition of Ti-Nb-Zr, along with its oxygen content, was determined to be 39.6 Nb, 4.3 Zr, 0.2 Fe, 0.002 wt.% O, with the balance being Ti. The difference between the nominal (37.5%) and actual (39.6%) contents of niobium in the processed alloy after ICP test is reasonable. The cast ingots were cut into slices using wire cut. Cold rolling of the slices was carried out by passing them separately through rollers at room temperature to improve their surface finish, enhance their mechanical properties, and continuously reduce their thickness (0.05 mm reduction with each rolling pass) to obtain a final thickness of 200 µm (a total reduction of 95%) (Figure 1b).

Cold-rolled specimens were then heat treated under argon atmosphere at different temperatures of 700, 850, and 900 °C for 30 min, followed by quenching in iced water. The specimens were then cut into different dimensions as reported in Table 1 for further characterization tests. In the present investigation, commercially pure titanium (cp-Ti, grade 1, 99.99%, Exotech, Pompano Beach, FL, USA) received no treatment as the control group to be compared with other groups.

### 2.2. Surface Characterization

Samples were ground up to 2000-grit finish with emery papers, followed by cloth polishing using a 20 nm colloidal silica suspension. After ultrasonic cleaning of the polished samples in analytical-grade acetone at room temperature for 30 min using a Powersonic 405 unit, samples were washed with ethanol and dried with an air blower. Before optical microscopy, samples were etched in a solution of 5 HF, 10 HNO_3_, and 85% H_2_O. Ultrasonic cleaning of specimens was performed sequentially in acetone, ethanol, and distilled water, each for 10 min.

Microstructural and chemical analysis of the surfaces were performed using optical microscopy (OM, Nikon, Epiphot 300, Tokyo, Japan), FESEM (QUANTA FEG-450, FEI, Lausanne, Switzerland) equipped with energy dispersive spectroscopy (EDS, Octane Elite, AMETEK, Berwyn, PA, USA). X-ray diffractometry (XRD, Asenware AW-DX300 with Cu-K*α* radiation) was used for phase analysis under accelerating voltage and current of 40 kV and 30 mA, respectively. Identification of unknown crystalline phases was further approved using the FEI Tecnai F20 S-Twin 200 kV (FEI, Lausanne, Switzerland) transmission electron microscope (TEM) with selected area electron diffraction (SAED) pattern. Before TEM, thinning of samples to electron transparency using a twin-jet electropolisher at a temperature of −35 °C in an electrolyte containing methanol, perchloric acid, and butyl glycol was performed. Vickers microhardness for each specimen was measured as the average of five measurements using a Buehler (Micromet 5101, Illinois) microhardness testing instrument, with an applied load of 200 g and a loading time of 10 s.

### 2.3. Cell Viability Assessment

A human osteoblast-like MG-63 cell line (c-555) was adopted from the National Cell Bank of Iran (NCBI) (Tehran, Iran) at Pasteur Institute to evaluate the cellular response of the specimens. After culturing cells in a culture medium containing 10% fetal bovine serum (FBS) and 1% penicillin/streptomycin for 24 h at 37 °C, 5000 MG-63 cells from the fourth passage were selected. After sterilization of specimens under UV light for 15 min, cells were seeded onto groups (*n* = 3) and cultured for 2, 4, and 7 days in a 48-well plate according to ISO standards 109930-12 at 37 °C with an atmosphere of 5 CO_2_ and 95% air. In vitro cytotoxic effects were evaluated using a 3-(4,5-dimethylthiazol-2-yl)-2,5-diphenyltetrazolium bromide (MTT, Sigma-Aldrich, Burlington, MA, USA) assay according to ISO 10993-5 standard. For this purpose, refreshing the culture medium every 2 days was performed. At each time interval, the culture medium was discharged, and 400 µL of a synthetic cell culture media (Minimum Essential Medium (MEM)) mixed with 40 µL of a yellow-colored MTT-labeled solution was used instead. Cell incubation continued for 3–4 h at 37 °C before measuring optical densities (ODs) at 540 nm by an Elisa-Reader (Bio-Tek Instruments Inc., Winooski, VT, USA). Positive and negative controls were considered cultured cells with no treatment.

### 2.4. Statistical Analysis

Statistical software (GraphPad Prism 10) was applied for cytotoxicity data analysis using the two-way analysis of variance (ANOVA test) [18]. MTT assays were done in triplicate, and the data were expressed as mean standard deviation (mean SD; *n* = 3). Tukey’s method was applied to compare between groups, with statistically significant differences being accepted at *p* < 0.05.

## 3. Results and Discussion

### 3.1. Morphological Features and Phase Analysis

**Figure 2:**
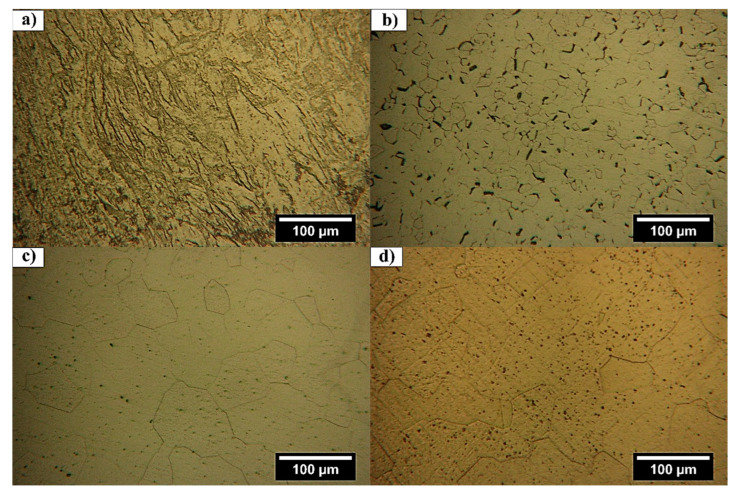
OM micrographs of Ti-Nb-Zr alloy after (**a**) cold rolling and heat treatment at (**b**) 700, (**c**) 850, and (**d**) 900 °C.

Optical micrographs of textural evolution during cold rolling and heat treatment processes are depicted in Figure 2. After cold rolling (Figure 2a), the formation of a lamellar (fibrous) structure consisting of elongated phases and grain boundaries, which typically appear in titanium [19], low carbon steels [20], and nickel/zirconium-based alloys [21], could be observed. Dislocation networks and stress-induced phases (e.g., *α*″ martensite, *ω*) induced by cold rolling [22,23] are present in OM (Figure 2a).

Heat treatment at 700 °C led to the formation of a uniform structure of the *β*-Ti phase with distinct grain boundaries consisting of dark, rod-like precipitates as secondary phases (Figure 2b).

**Figure 3:**
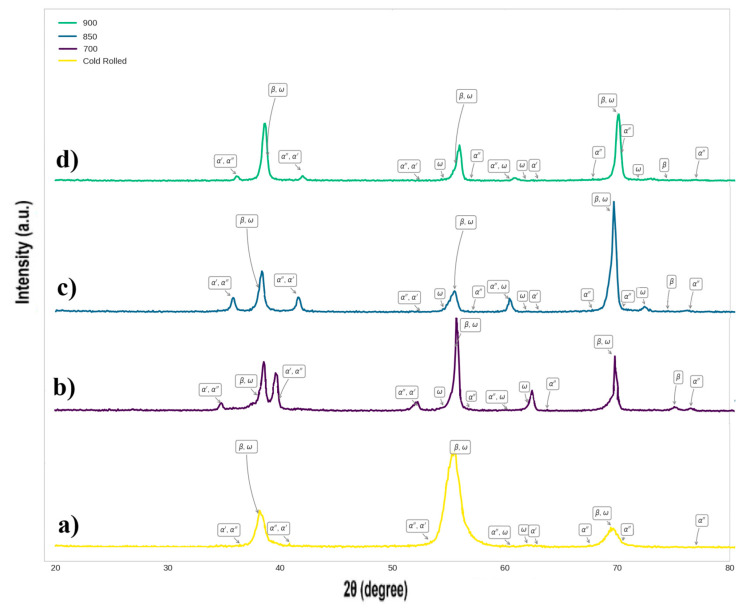
XRD patterns of Ti-Nb-Zr alloy specimens after heat treatment at (**a**) 700, (**b**) 850, (**c**) 900 °C, and (**d**) cold rolling.

**Figure 4:**
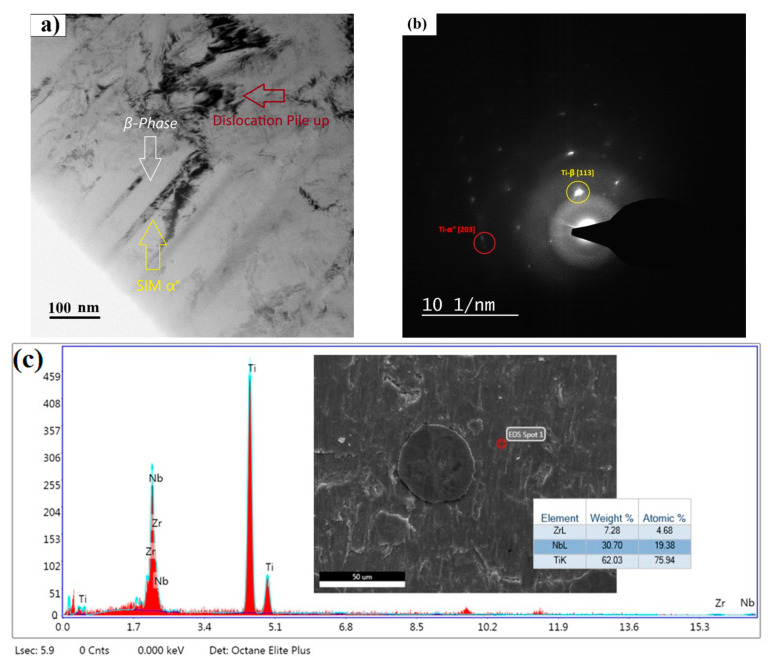
(**a**) TEM confirmation for co-existence of *α*″ and *β* phases in the matrix for cold-rolled Ti-Nb-Zr specimen, (**b**) SAED pattern corresponding to zone axis [113] of Ti-*β* and [203] of Ti-*α*″, confirming the dual-phase structure via diffraction spots, and (**c**) EDS spectrum (main plot) and corresponding SEM micrograph (inset) of a representative area, showing the chemical composition at EDS Point 1. The analysis indicates Ti, Nb, and Zr as the primary chemical constituents, with their respective weight and atomic percentages presented in the embedded table.

As confirmed by XRD results (Figure 3), black precipitates should be *ω*, *α*′ or *α*″ secondary phases, which usually appear in Ti-Nb-Zr alloys during the cold rolling process and rapid cooling [24,25,26], as represented by TEM results in Figure 4a.

At 700 °C heat treatment, the peaks of the martensitic *α*′ and *α*″ phases become much weaker, while the intensity of the *ω* phase significantly increases. This approves the transformation processes of martensitic phases to the *ω* phase (*α*′/*α*″ → *ω*) and the direct transformation of the *β* phase to the *ω* phase. Relatively small grain sizes indicate that no significant grain growth at a heat treatment temperature of 700 °C occurred due to a low diffusion rate (Figure 2b). By increasing the heat treatment temperature to 850 °C, grain boundaries remained distinct, and significant grain growth occurred (Figure 2c). At 850 °C heat treatment, the proportion of the *ω* phase decreased slightly, while intensities of the *α*′ and *α*″ phase peaks increased (Figure 3). The absence of rod-like precipitates in the structure indicates higher stability of the *β* phase [27]. Moreover, the presence of niobium as a *β*-stabilizer in titanium alloys reduces the martensitic start temperature (*M_s_*), enabling *β* to *α*″ transformation to occur at or near room temperature, thus activating TRIP effects under mechanical loading [28]. In this regard, TRIP involves stress- or strain-induced transformations of the *β* phase into the orthorhombic *α*″ martensitic phase, which enhances ductility and work hardening by dynamically restricting dislocation motion through phase interfaces and twin boundaries [29]. In addition to niobium, zirconium addition is regarded as a *β*-phase stabilizer and beneficially suppresses the formation of the detrimental *ω* phase, typically associated with embrittlement and reduced ductility in *β* titanium alloys, upon quenching from high temperatures. On the other hand, activation of TWIP mechanisms in the presence of Zr contributes to improved ductility and mechanical performance in metastable *β* titanium alloys [6]. This activates multiple TWIP deformations (including {332}<113>, {112}<111>, and {5 8 11}<135> twinning systems), which enhance ductility and strain-hardening rate more effectively than dislocation slip alone [30].

By increasing the heat treatment temperature to 900 °C (Figure 2d), the grains grew even larger than those of the specimen at 850 °C (Figure 2c), with more ductility changes in grain boundaries. This was reasonable due to an increase in diffusion rate with heat treatment temperature. At 900 °C heat treatment, although the *β* phase is maintained stable upon quenching, very fine precipitates of the *ω* phase were formed. The very small size of the precipitates proves the stoppage of phase transformation reactions at very early stages due to rapid quenching. However, the number of precipitates at 900 °C is higher than that at 850 °C.

### 3.2. Mechanical Properties

**Figure 5:**
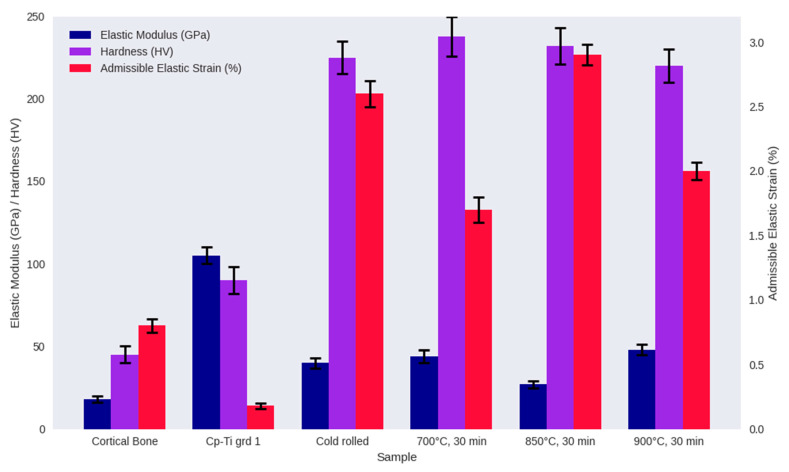
Mechanical properties of the specimens vs. cp-Ti as the control. Elastic admissible strain is defined as the fraction of yield strength to elastic modulus.

Mechanical properties of alloy specimens in comparison with commercially pure titanium (cp-Ti) as a control and cortical bone as a reference are illustrated in Figure 5. It seems like alloying elements of Nb and Zr appropriately help in reducing the mismatch between the elastic modulus of pure titanium and cortical bone. A superior combination of mechanical properties, attributed to the synergistic effects of phase precipitation strengthening and the harmonious interaction of the TWIP/TRIP mechanisms, could be observed for alloyed specimens compared with pure titanium.

Heat treatment was successful in balancing hardness (220–250 Hv) and low modulus (25–50 GPa) by minimizing harmful phase interactions through transforming stress-induced *α*″/*ω* phases into a uniform *β* phase structure and modulating dislocation-driven *α*-phase precipitation at grain boundaries. The stabilized *β* phase maintains mechanical compatibility with bone (admissible strain ~1.3%), which represents greater suitability of the proposed material for biomedical implant applications [31,32,33,34,35].

### 3.3. Cell Viability

**Figure 6:**
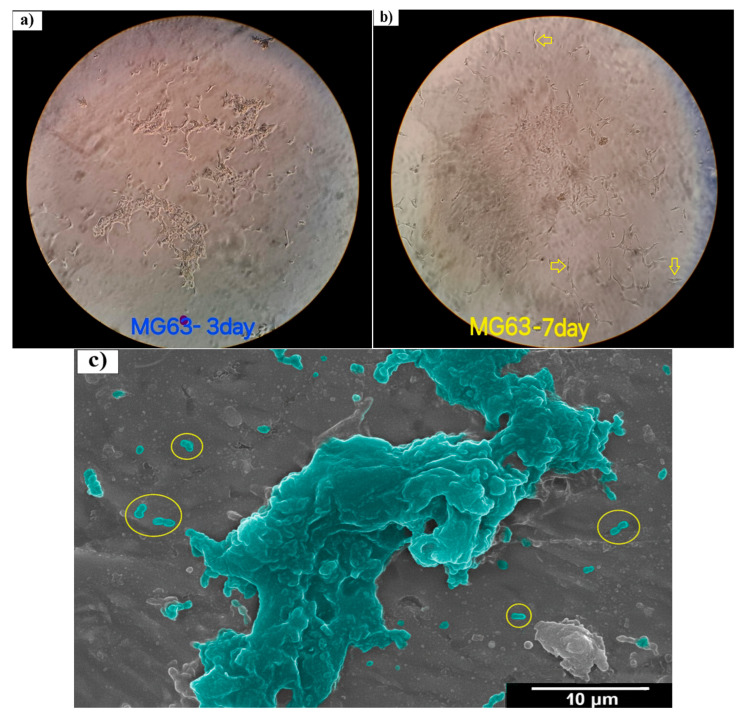
Overall morphology of MG-63 cells (**a**) cultured in culture flask for 3 days, (**b**) cultured in culture flask for 7 days, and (**c**) on the surface of cold-rolled Ti-Nb-Zr alloy for 7 days.

Osteoblast-like MG-63 cells were cultured in the culture flask to assess their metabolic activity. Microscopic observation of cell morphology and proliferation was performed on cells cultured for 3 and 7 days, as represented in Figure 6a,b. The cells exhibited a spindle-shaped morphology (arrows and circles in Figure 6b,c) and fused closely into a monolayer during culture time. Stages of cell division and proliferation were also detected on SEM images of samples co-incubated with MG-63 cells for 7 days, as represented in Figure 6c. A large number of adherent cells were observed after 7 days. The spindle-shaped morphology of the cells is visible (circles with yellow borders) in Figure 6.

**Figure 7:**
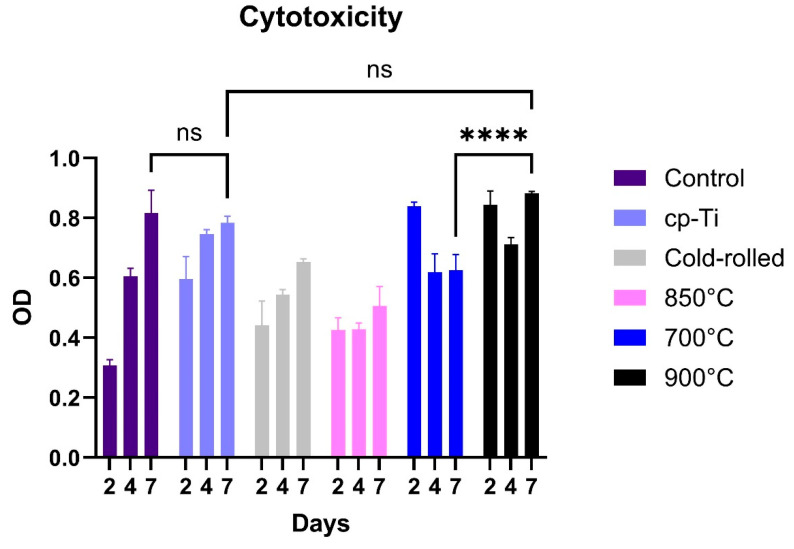
Comparative viability of MG63 cells seeded on cp-Ti and Ti-Nb-Zr alloy in its cold-rolled and heat-treated states after 2, 4, and 7 days of incubation. Data are represented as mean (*n* = 3) ± SD values. Significant difference (**** *p* < 0.0001) is observed. ns—not significant.

MTT results for MG-63 cells after 2, 4, and 7 days of direct exposure with cp-Ti and the designed Ti-Nb-Zr alloy in its cold-rolled/heat-treated states are expressed in Figure 7. The absorbance (OD value) for the Ti-Nb-Zr alloy after cold rolling and different heat treatment temperatures is different. This is because strain-induced transformations can influence cell viability in Ti-Nb-Zr alloys by altering surface properties and mechanical stability [4,6,35]. Overall, it can be observed that heat treatment at 850 °C has degraded the cell response of the alloy with respect to its cold-rolled state, and both show viabilities lower than those recorded for the control well and cp-Ti.

In the case of cp-Ti, despite the fact that it is non-cytotoxic and supports cell adhesion, this alone is not enough to explain its higher absorbance over 2 days and 4 days of co-incubation compared with the control well. One possible reason could be that the control well has fewer cells since they are detached from the surface due to poor adhesion. In this regard, the biocompatibility of cp-Ti could result in comparatively higher metabolic activity. Cell viabilities of the alloy in its cold-rolled state are lower than those of the cp-Ti sample. This is because cold rolling imparts substantial plastic strain to the alloy, reducing the stability of the *β* phase and facilitating strain-induced martensitic (SIM) transformation of *β* to *α*′/*α*″ as previously represented in Figure 4a.

After 2 days of MTT assay, significant cell viability of the sample heat-treated at 700 °C could be attributed to the presence of *ω* precipitates, which help to stabilize interfaces between different transformed phases and contribute to the alloy’s structural integrity [36,37,38,39,40]. On the contrary, the decrease in absorbance of this sample over 4 days should not necessarily be due to any cytotoxic effect since the absorbance remains constant over 7 days. Considering the high absorbance of this sample after 2 days, as high as the absorbance of the control well after 7 days, a nutrient depletion or waste accumulation could be one possible reason for this lowering of metabolic activity without cell death.

At 850 °C heat treatment, the decrease in cell viability could be attributed to the decrease in the *ω* phase, compared with the sample heat-treated at 700 °C (Figure 7), which indicates greater thermodynamic instability of this sample compared with 700 °C. Moreover, treatment of a sample heat-treated at 850 °C resulted in the formation of a stable, non-proliferating cell population with constant absorbance within the first 4 days, followed by an increase at day 7. This consistency is possible since the MTT assay does not rely on absolute cell numbers but reflects mitochondrial reductase to convert tetrazole to formazan, which is dependent on the number of viable cells.

At 900 °C, with respect to Figure 3, the peaks of the *β* phase begin to increase in intensity, gradually becoming the dominant phase in the structure. This explains the significantly higher cell viability of alloy heat-treated at 900 °C compared with 700 °C (Figure 7). Moreover, the drop in absorbance of this sample from day 2 to 4 should not be regarded as cytotoxicity, as it soars again over 7 days to an absorbance significantly higher (Figure 7, *p* < 0.0001) than that recorded for the sample heat-treated at 700 °C. This indicates an increased enzymatic activity without actually having an effect on cell number or cell viability. Biocompatibility of the alloy heat-treated at 900 °C is not significantly higher compared with cp-Ti over 7 days of co-incubation.

**Figure 8:**
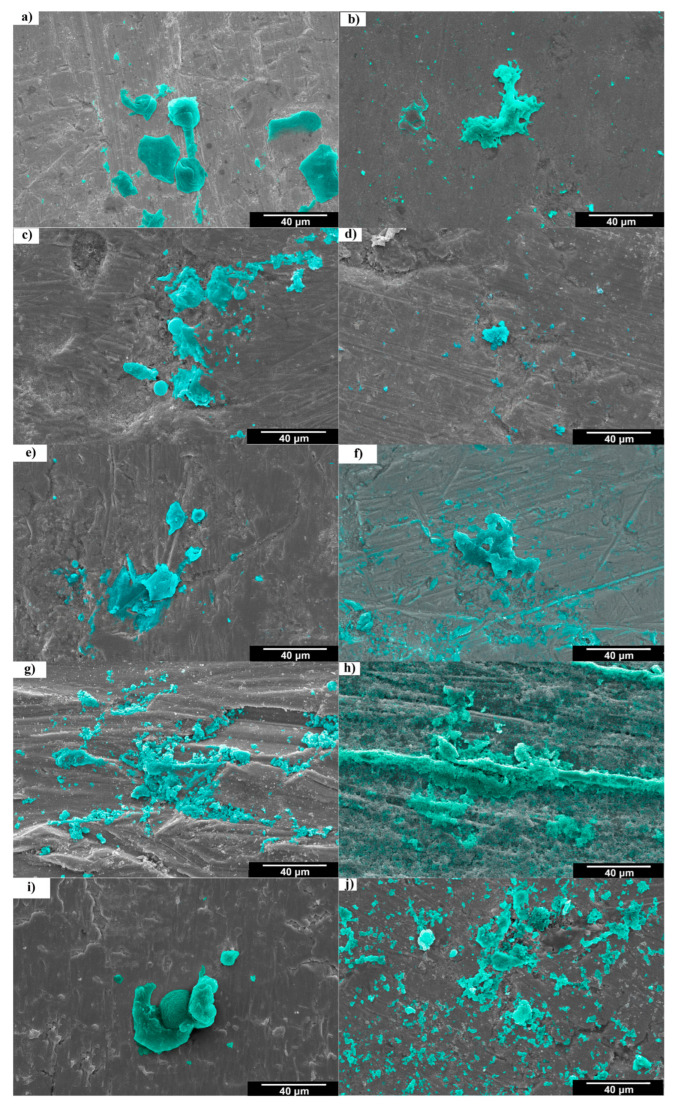
SEM images of MG-63 cells (colored in blue) distribution and shapes after 2 days (**a**,**c**,**e**,**g**,**i**) and 7 days (**b**,**d**,**f**,**h**,**j**) of MTT assay on alloys heat treated at 850 (**a**,**b**), 700 (**c**,**d**), 900 °C (**e**,**f**), commercially pure titanium (**g**,**h**), and cold-rolled alloy before heat treatment (**i**,**j**).

SEM images (Figure 8) show cytocompatibility of attached MG-63 cells on the surface of alloys and pure titanium after 2 and 7 days. After being cultured for 2 days, the initial adhesion of MG-63 cells in the form of sparse spheres on the surface of alloys was confirmed. In the case of pure titanium, spreading cells with some filopodia together with round-shaped cells were observed after 2 days (Figure 8g). After 7 days of incubation, cell proliferation increased in all samples, but it appeared significantly as dense, lamellar, and confluent networks spreading on the surface of pure titanium (Figure 8h), indicative of the advanced stage of cell adhesion. Alloys heat-treated at 700 and 900 °C represented spreader cell morphologies similar to those of pure titanium but with the lower density of networks (Figure 8d,f).

Implants placed in the body, such as dental implants, are subjected to repeated loads like chewing. If the implant material reaches its yield strength and undergoes permanent deformation, it can lead to loosening, misalignment with the bone, and ultimately implant failure. The implant must be able to maintain its mechanical function long-term without deforming. Plastic deformation over time can lead to the loss of integrity between the implant and its surrounding tissue.

**Figure 9:**
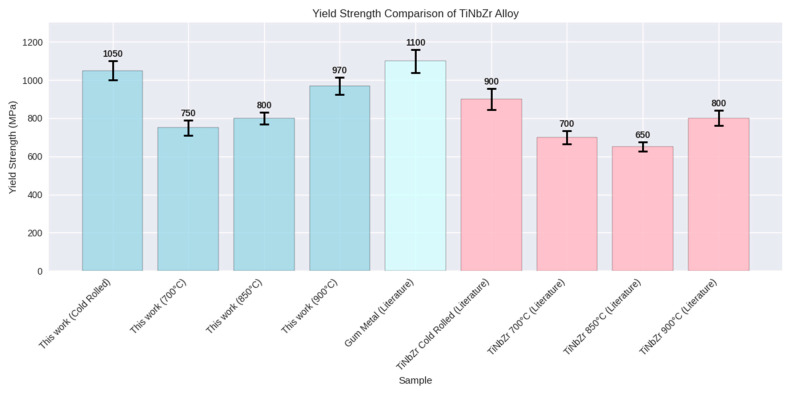
Yield strength comparison of a novel Ti- 37.5 Nb- 4.5 Zr (wt.%) alloy from this work with literature values for Ti- 23 Nb- 0.7 Ta- 2 Zr- 1.2 O gum metal [9,11], Ti-Nb-Zr-Ta-Hf concentrated alloys [41], Ti-Nb-Zr-Fe-O gum-type alloy [42], and Ti-Nb-Fe alloys with Zr/Sn additions [43].

According to Figure 9, the yield strength of the alloy in this study, both in the cold-rolled state and after heat treatment at various temperatures (700, 850, and 900 °C), is generally higher than typical Ti-Nb-Zr values reported in scientific literature. However, in the cold-rolled state, it remains lower than gum metal. This comparison indicates that the alloy in this research shows good potential, especially at 900 °C, where there is a significant difference compared with the references [9,11,41,42,43].

## 4. Conclusions

This study investigated the effects of plastic deformation mechanisms and phase evolutions on cell viability performance of a novel Ti-Nb-Zr alloy for biomedical applications. Our results revealed a clear link between heat treatment temperature and changes in phase structure as well as cell response of the designed alloy. In its cold-rolled state, the alloy structure consisted of a large portion of unstable *β* phase along with stress-induced *α*″ and *ω* martensitic phases, which all negatively affected its cell viability. Upon gradually increasing the heat treatment temperature from 700 to 900 °C, the predominance of the stabilized *β* phase helped to minimize detrimental phase interactions and improve cell proliferation results. Microstructures formed by controlled heat treatment activated TRIP and TWIP deformation mechanisms and led to an excellent combination of mechanical properties. Specifically, the heat-treated Ti-Nb-Zr alloy showed a much lower elastic modulus (25–50 GPa) and a higher elastic admissible strain (around 1.3%), making it mechanically compatible with human bone. This optimized mechanical behavior, along with significantly improved MG-63 cell viability (especially at 900 °C), strongly supports the potential of this Ti-Nb-Zr alloy as a promising candidate for next-generation orthopedic and dental implants.

## Figures and Tables

**Table 1:** Dimensional details of the samples prepared for different characterization tests.

Test	Specimen Shape	Dimensions (mm)
Optical microscopy (OM)	Plate	10 × 10 × 0.25
Vickers micro hardness	Flat surface from sheet	10 × 10 × 0.25
X-ray diffraction (XRD)	Flat-cut plate	10 × 10 × 0.25
Transmission electron microscopy (TEM)	Thin disc	Diameter: 3
		Thickness < 0.1
Cell viability assessment	Plate	5 × 5 × 0.25
Field emission scanning electron microscopy (FE-SEM)	Rectangular plate	5 × 5 × 0.25
Uniaxial tensile test	Dog-bone/rectangular	Gauge length: 15
		Width: 3
		Thickness: 0.25
		Total length: 45
		Fillet radius: 3
		Width of ends: 5

## Data Availability

The original contributions presented in this study are included in the article. Further inquiries can be directed to the corresponding author.
